# Prevalence and factors associated with rotavirus diarrhea among children aged 3–24 months after the introduction of the vaccine at a referral hospital in Uganda: a cross-sectional study

**DOI:** 10.1186/s12887-024-04842-8

**Published:** 2024-05-23

**Authors:** Goretty Laker, Jolly Nankunda, Bernis Maren Melvis, Dickson Kajoba, Martin Nduwimana, Joel Kimera, Richard Justine Odong, Isaac Edyedu

**Affiliations:** 1 Department of Pediatrics and Child Health, Kampala International University, Kampala, Uganda; 2 Department of Surgery, Kampala International University, Kampala, Uganda; 3 Mulago specialised Women and Neonatal Hospital, Kampala, Uganda

**Keywords:** Diarrhea, Low income country, Rotavirus, Uganda

## Abstract

**Background:**

Rotavirus has a significant morbidity and mortality in children under two years. The burden of rotavirus diarrhea 4 years post introduction of rotavirus vaccine in Uganda is not well established. This study aimed to determine the prevalence, severity of dehydration and factors associated with rotavirus diarrhea among children aged 3 to 24 months after the introduction of the vaccine at Fort Portal Regional Referral hospital.

**Methods:**

This was a cross-sectional hospital-based study in which children with acute watery diarrhea were included. A rectal tube was used to collect a stool sample for those unable to provide samples. Stool was tested for rotavirus using rapid immunochromatographic assay. Data was analysed using SPSS version 22 with logistic regression done to determine the factors.

**Results:**

Out of 268 children with acute watery diarrhea, 133 (49.6%) were females. Rotavirus test was positive in 42 (15.7%), majority of whom had some dehydration 28(66.7%). The factors that were independently associated with rotavirus diarrhea were; age < 12 months (AOR = 8.87, *P* = 0.014), male gender (AOR = 0.08, *P* = 0.001), coming from a home with another person with diarrhea (AOR = 17.82, *P* = 0.001) or a home where the water source was a well (AOR = 50.17, *P* = 0.002).

**Conclusion:**

The prevalence of rotavirus diarrhea was three times less in the post rotavirus vaccination period compared to pre-rota vaccination period. Majority of the participants with rotavirus diarrhea had some dehydration. There is need for provision of safe water sources to all homes. Surveillance to determine the cause of the non rota diarrhea should be done.

## Background

The rotavirus is a double-stranded RNA virus that belongs to the Reoviridae family [1]. Rotavirus is composed of three concentric shells that enclose 11 gene segments [1]. The outermost shell contains two proteins (VP7 0R G-protein and VP4 or P-protein) which are believed to be involved in immune protection and they are responsible for the classification of the rotavirus [1]. The rotavirus genome segments encodes 6 structural proteins which make up virus particles (Viral protein or VP) and 6 non-structural proteins (NSP). The type of rotavirus detected in this study was rotavirus A [1].

Children between the ages of 3 and 24 months experience the most symptomatic illness [1]. Rotavirus diarrhea is due to rotavirus infecting and destroying the enterocytes bringing about malabsorption as the chief mechanism of diarrhea. Rotavirus also induces intestinal secretions through activation of the enteric nervous system and also leads to antigenemia which is associated strongly with manifestation of acute gastroenteritis [2].

Rotavirus the primary cause of diarrheal illness worldwide is projected to result in 2.4 million hospitalizations and 114 million episodes per year requiring home care [3]. Although there have been reports of rotavirus spreading through water among young children, rotavirus is typically spread from person to person by the oral-fecal route [4, 5]. The most common cause of severe acute diarrhea in the world, rotavirus has a significant morbidity and fatality rate in children under the age of five [2].

Globally, rotavirus infection was the leading cause of diarrheal deaths, accounting for 19.11% of deaths from diarrhea in 2019. Rotavirus has caused a higher death burden in African, Oceanian, and South Asian countries in the past three decades [6]. Sub-Saharan Africa contributed 121,000 deaths of 215,000 rotavirus related deaths globally in 2016 [6]. As a result, it was listed as the third most common infection linked to mortality in children under the age of five [7].

While there are many different laboratory techniques for diagnosing rotavirus, enzyme-linked immunosorbent tests and quick stool antigen detection assays with reverse transcription polymerase chain reaction are frequently utilized, particularly in research laboratories [5, 8]. The identification and classification of dehydration status forms the foundation of care and is crucial to the evaluation of acute diarrhea [9]. Dehydration status is classified as: no dehydration, some dehydration, or severe dehydration, and is therefore treated according to plans A, B, and C respectively [10].

As of 2018, more than 92 nations had integrated vaccines into their national immunization programs, following WHO recommendations to utilize 2 rotavirus vaccines in all national vaccination programs worldwide since 2006 [11]. Uganda introduced the rotavirus vaccine (Rotarix) a 2 dose vaccine into the routine immunization schedule in June 2018, however global supply challenges in 2022 through 2023 necessitated the switch to a new formulation to ensure that children continue to be protected from rotavirus disease [6]. On the impact of the introduction of rotavirus vaccination on diarrheal fatalities in Africa, there are no any post-rotavirus vaccination population-based data available [12].

Diarrhea in Uganda is still ranked among the top 10 causes of morbidity, mainly because it is highly contagious and associated with severe dehydration leading to shock and multiorgan failure [13, 14]. Studies carried out at Mulago National Hospital in 2010 and 2012 respectively found a pre-rotavirus vaccination prevalence of 45.4% among children aged 3–59 months [14] and 32.8% among children under 5 years [15]. However, there is a paucity of data on post-vaccination diarrhea status in the country since its rollout in 2018. This study was done to determine the prevalence of rotavirus, severity of dehydration and factors associated with rotavirus diarrhea among children aged 3 to 24 months fours post introduction of the vaccine at Fort Portal Regional Referral Hospital.

## Methods

### Study design and setting

This was a hospital based cross sectional study done at Fort Portal Regional Referral Hospital (FRRH) in the Pediatric department (OPD, ward and Nutrition unit) from December 2022 – February 2023. FRRH is one of the public regional referrals under the Ministry of health in western Uganda that also serves as a Teaching Hospital for Kampala International University. It is a specialized hospital with specialized services in pediatrics and child health. The pediatric ward has a seventy five-bed capacity. The pediatric ward, OPD and emergency is managed by four pediatricians, three senior housing officers and seven nurses. At Fort Portal Regional Referral Hospital, acute diarrhea is managed according to the WHO guideline [16].

### Study population

All children 3–24 months old who presented with diarrhea at Fort Portal Regional Referral hospital for medical care during the study period were eligible. Three months was the lower limit because at this age rotavirus immunization is complete in Uganda. The upper limit of 24 was chosen because 3–24 months marks the peak of rotavirus infection [15, 17].

### Eligibility criteria

Inclusion was for children with acute watery diarrhea aged 3–24 months whose caregivers consented. Children with persistent or chronic diarrhea were excluded.

### Sample size and sampling

Using open Epi software for calculating sample size for 2 proportions, a sample considering an odds ratio of 2.0 for occurrence of rotavirus infection among children with acute diarrhea in Kenya [17], considering two-sided confidence level (1- alpha) = 95%, Power = 80%, ratio of controls to cases = 1, least extreme odds ratio to be detected = 2.00, giving a total sample size of 268 participants. Consecutive sampling technique was used until the desired sample size was obtained.

### Data collection procedure

At the outpatient clinic of FRRH, patients were triaged for participation. Those admitted were identified using the ward in-patient registers. Criteria for admission were; excessive vomiting associated with some dehydration, severe dehydration and children with acute malnutrition admitted for inpatient therapeutic feeding. Those who did not meet the admission criteria were included in the study from the outpatient department.

After identifying the study participants, time was spent to explain the study protocols in simple language and then consent was sought. For those who provided informed consent, the researcher went ahead and administered the questionnaire to collect information on diarrhea, social demographics, child factors and family social factors. Further the researcher examined the participants for dehydration signs and after which a rectal tube was used to collect the stool sample for rotavirus testing for those unable to provide stool samples.

Those found to have rotavirus were managed as a case for viral diarrhea; antibiotics were stopped for those who were taking them. They were given zinc tablets, and managed according to their level of dehydration. They were advised to continue feeding. They were also advised on the importance of hand washing, and they were encouraged to boil water for drinking.

### Data collection instruments

A semi-structured questionnaire with closed ended questions was used to obtain social demographic, child and family social factors associated with rotavirus diarrhea, hydration status and rotavirus test result. This was administered and filled by the principal investigator and research assistant. Every patient who presented with acute diarrhea was evaluated for dehydration status and was further classified as either no dehydration, some dehydration or severe dehydration according to the WHO classification [16].

### Rotavirus testing procedure

This study adopted the methodology used by Sharma (2017) [18] in his study “comparison of a rapid immunochromatography test with Elisa to detect rotavirus”. Sample collection was done by means of a rectal tube for those unable to provide stool samples. A 5 ml syringe was attached to a rectal tube size 10 which was inserted into the rectum following aseptic technique [14]. The tube was inserted approximately 2 cm in the rectum while the child was held on the mother’s thighs in prone position and 5mls of stool was aspirated. The sample was placed into a sterile and dry screw-top stool container. The container was labelled with a unique identifier of the study participant.

Assay diluent was taken in a disposable dropper up to the line marked on it and then transferred into the sample collection tube. This was done twice. Sample collection swab was put into the stool sample and then inserted into the tube containing assay diluent (sample collection tube) [18]. Sample collection swab was then swirled ten times in the sample collection tube until the sample dissolved into the assay diluents. The swab was discarded while squeezing it against the wall of tube. The presence of only control band within the result window indicated a negative result [18]. The presence of two-color bands as test band (T) and control band (C) within the result window, no matter which band appeared first, indicated a positive result and having only the presence of the test band was considered invalid [18].

The study used immunochromatographic assay (Fastep kit sensitivity- 90%, specificity- 93%) for the diagnosis of rotavirus diarrhea. This was because this test is easy to perform, it provides rapid results and has a high sensitivity [1]. A study in tertiary Care Hospital in Bangladesh showed immunochromatographic assay sensitivity of 90.70% and specificity of 93.88% in comparison to ELISA [19] while in India it had a sensitivity of 95.24% and specificity of 97.47% [20]. The kit employed in this study used the lateral flow method, took 10–15 min to give results, required fecal specimen and was manufactured by polymed therapeutics inc. Houston, USA. Registration number 4,003,234. Rapid immunochromatographic assay was carried out according to procedure provided by the manufacturer.

### Quality assurance

Data quality was assured by collecting data from caregivers of the children who met the inclusion criteria, after stabilizing the unstable patients. The questionnaire was pretested for reliability and validity prior to the commencement of data collection. Research assistants were trained nurses who were trained on how to identify signs of dehydration, collection of stool sample and how to fill the questionnaires. The principal investigator had training on how to carry out the rapid diagnostic test of rotavirus using the immunochromatography by laboratory technologist before commencement of the study. For every 20 samples, one sample was taken to a reference laboratory to check for consistence of the result findings. Each filled questionnaire was cross-checked for consistencies and completeness before the interview was terminated.

### Data analysis

Data was entered in Microsoft excel software, cleaned and sorted and thereafter exported to SPSS version 22. To determine the prevalence of rotavirus diarrhea, univariate analysis was done and presented as a pie chart in percentages. To classify the dehydration status of rotavirus diarrhea, univariate analysis was done and presented in a tabular form as frequencies and percentages. To identify factors associated with rotavirus diarrhea, analysis was done at both bivariate and multivariate. At bivariate level, crude odds ratios and *P*-values were computed and presented in a table with significance set at 95% and any variable with *p*-value less than 0.2 was subjected to multivariate logistic regression to establish associations presenting adjusted odds ratio and *P*-value in tables with significance set at 95% (*P*-value < 0.05).

### Ethical considerations and consent

All methods were carried out in accordance with relevant guidelines and regulations. Ethical approval was sought from the Research and Ethics Committee of Kampala International University Western Campus (**Ref No: KIU-2022-188)**. All parents/guardians gave written informed consent.

## Results

**Fig. 1:**
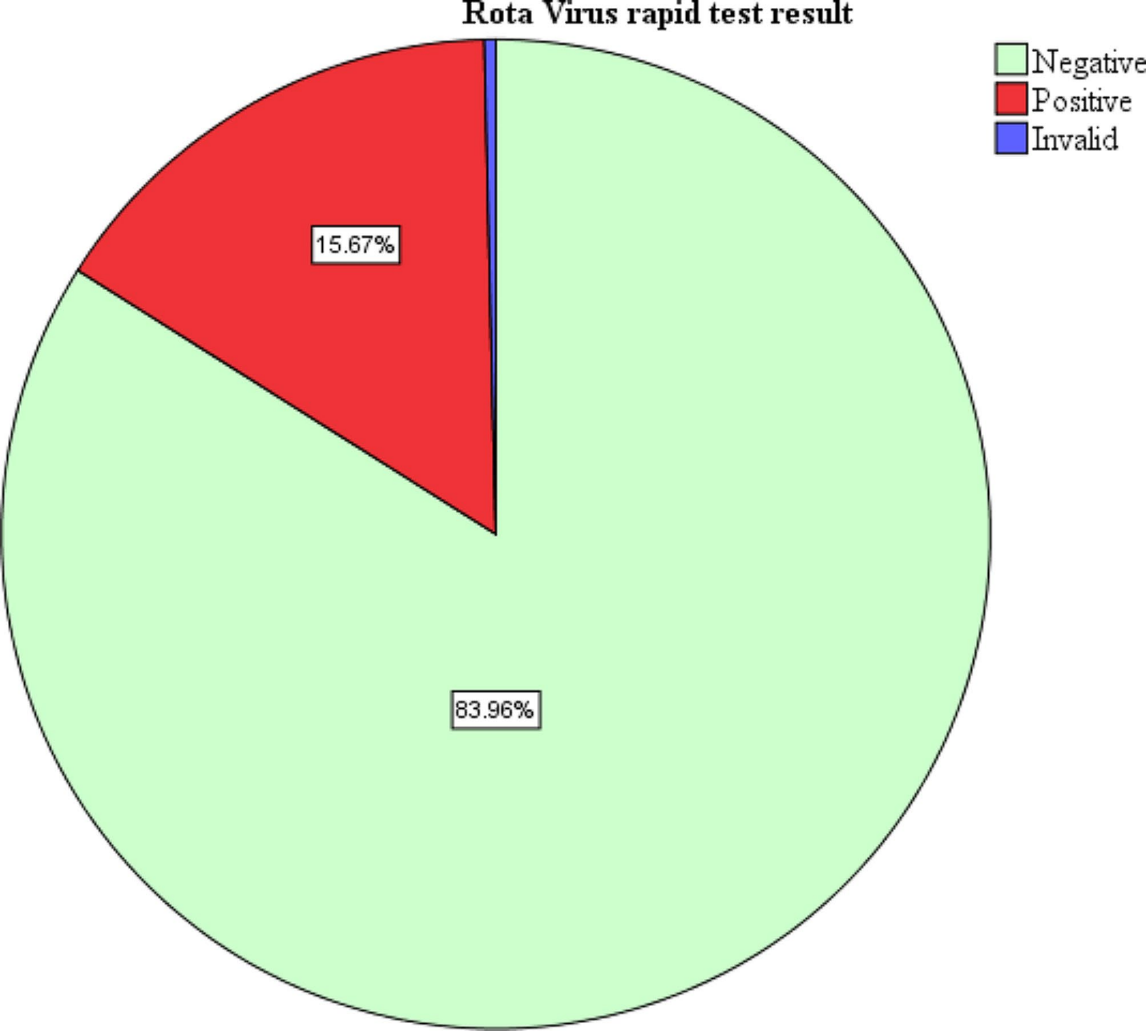
Prevalence of Rota-virus Diarrhea among children aged 3–24 months old attending Fort Portal Regional Referral hospital

During the study period, 268 children with diarrhea were enrolled. All the children enrolled had the rapid test for rotavirus done. About half of the children enrolled with diarrhea were females 135(50.4%). Majority of the participants were aged 3–12 months 177 (66.0%). Majority of the participants had diarrhea for less than 5 days 220 (82.1%). In this study, one test result was invalid. Forty-two tests were positive representing a prevalence of 15.7% (Fig. 1). Among the study participants with rotavirus diarrhea, majority had some dehydration 28(66.7%) while only 2 children (4.8%) with severe dehydration (Table 1).

**Table 1:** Dehydration status in children aged 3–24 months with diarrhea attending Fort Portal Regional Referral hospital

Dehydration status	rotavirus Positive	rotavirus Negative	Total
No Dehydration	12 (28.6%)	169 (75.1%)	181(67.8%)
Some dehydration	28 (66.7%)	54 (24.0%)	82 (30.7)
Severe dehydration	2 (4.8)	2 (0.9)	4(1.5)
Total	42 (100.0)	225 (100.0)	267(100.0)

At multivariate level of analysis, the variables that were significantly associated with rotavirus infection were child’s age, gender, having another child with diarrhea at home and getting water from a well. In this study, a child aged less than 12 months was 8.87 times more likely to have rotavirus compared to one older than 12 months (AOR = 8.87, CI = 1.55–50.74, *P* = 0.014). A male child was 0.083 times less likely to have rotavirus infection compared to a female (AOR = 0.08, CI = 0.02–0.36, *P* = 0.001). A child from a home with another person with diarrhea was 17.82 times more likely to have rotavirus infection (AOR = 17.82, CI = 3.48–91.17, *P* = 0.001). A child from a home where water for home use was obtained from a well was 50.17 times more likely to have rotavirus infection compared to one from a home where water was obtained from a tap (AOR = 50.17, CI = 4.40-71.97, *P* = 0.002). The details of bivariate and multivariate analysis are shown in Table 2. It is also important to note that 15% of the children reported as completely vaccinated had rotavirus.

**Table 2:** Bivariable and multivariate analysis of factors associated with Rotavirus diarrhoea

			Bivariate			Multivariate		
Characteristic	Rotavirus		COR	95% CI	*P* value	AOR	95% CI	*P* value
	Negative, *N* (%)	Positive, *N* (%)						
**Child’s age (months)**								
3–12	142(80.7)	34(19.3)	2.48	1.09–5.62	0.029	8.87	1.55–50.74	0.014*
13–24	83(91.2)	8(8.8)	1					
**Sex**								
Male	119(90.1)	13(9.9)	0.39	0.20–0.81	0.011	0.08	0.02–0.36	0.001*
Female	106(78.5)	29(21.5)	1					
**Mother’s age (years)**								
≤ 20	52(92.9)	4(7.1)	0.25	0.07–0.85	0.027	0.07	0.01–1.67	0.120
21–30	137(83.5)	27(16.5)	0.65	0.29–1.42	0.277	0.19	0.04–1.89	0.135
> 30	36(76.6)	11(23.4)	1					
**Mother’s education**								
None	29(90.6)	3(9.4)	0.37	0.09–1.49	0.161	0.02	0.01–1.48	0.117
Primary	90(81.1)	21(18.9)	0.83	0.34-2.00	0.677	0.63	0.08–4.81	0.651
Secondary	74(89.2)	9(10.8)	0.43	0.16–1.19	0.105	1.22	0.20–7.64	0.832
Tertiary	32(78.0)	9(22.0)	1					
**Still breast feeding**								
Yes	153(82.3)	33(17.7)	1.73	0.78–3.80	0.175	5.66	0.97–33.11	0.054
No	72(88.9)	9(11.1)	1					
**Exclusive BF duration**								
< 6	101(84.2)	19(15.8)	1.01	0.52-2.00	0.967			
6	124(84.3)	23(15.7)	1					
**Rota Immunization**								
None	10(83.3)	2(16.7)	1					
Partial	49(89.1)	6(10.9)	0.61	0.11–3.48	0.580			
Complete	90(84.9)	16(15.1)	0.89	0.18–4.44	0.886			
Not sure	76(80.9)	18(19.1)	1.18	0.24–5.88	0.836			
**HIV status**								
Negative	123(87.2)	18(12.8)	1					
Exposed	18(66.7)	9(33.3)	3.42	1.33–8.75	0.010			
Positive	3(100)	0(0.0)	N/A					
un known	81(84.4)	15(15.6)	1.27	0.60–2.65	0.533			
**Duration of diarrhoea (days)**								
< 5	183(83.2)	37(16.8)	1					
5–7	38(88.4)	5(11.6)	0.65	0.24–1.76	0.398			
> 7	4(100)	0(0.0)	N/A					
**Number of under-fives at home**								
None	75(79.8)	19(20.2)	1					
1–2	125(86.2)	20(13.8)	0.63	0.32–1.26	0.192			
> 2	25(89.3)	3(10.7)	0.47	0.13–1.74	0.260			
**Other person with diarrhoea at home**								
Yes	16(55.2)	13(44.8)	5.86	2.56–13.41	< 0.001	17.82	3.48–91.17	0.001*
No	209(87.8)	29(12.2)	1					
**Number of people at home**								
< 3	92(81.4)	21(18.6)	1					
3–5	87(90.6)	9(9.4)	0.45	0.20–1.04	0.063	0.03	0.01–1.20	0.101
> 5	46(79.3)	12(20.7)	1.14	0.52–2.53	0.741	0.08	0.01–1.50	0.107
**Bread winner’s occupation**								
Peasant	93(78.8)	25(21.2)	1.02	0.45–2.33	0.960			
Business	94(93.1)	7(6.9)	0.28	0.10–0.80	0.017			
Formal employment	38(79.2)	10(20.8)	1					
**Water Source**								
Tap	115(91.3)	11(8.7)	1					
Borehole	50(89.3)	6(10.7)	1.26	0.44–3.58	0.672	6.33	0.99–40.67	0.052
Protected spring	36(78.3)	10(21.7)	2.90	1.14–7.39	0.025	3.70	0.62–22.13	0.151
Well	24(61.5)	15(38.5)	6.53	2.67–15.97	< 0.001	50.17	4.40-71.97	0.002*
**Drink Boiled water**								
Yes	170(90.9)	17(9.1)	1					
No	55(68.7)	25(31.3)	4.55	2.29–9.040	< 0.001	3.57	0.29–9.040	0.061
**Toilet Type**								
Pit latrine	154(92.2)	13(7.8)	1					
VIP Toilet	20(71.4)	8(28.6)	4.74	1.75–12.83	0.002	3.75	0.75–12.83	0.072
Mud and wattle	51(70.8)	21(29.2)	4.88	2.28–10.44	< 0.001	5.86	0.28–10.44	0.091

COR = Crude odds ratio, CI = Confidence interval, N/A = Not applicable, AOR = Adjusted odds ratio. *significant

## Discussion

The study finding established a post rota vaccination era prevalence of 15.67% among infants 3–24 months of age attending Fort Portal Regional Referral Hospital. This study represents a reduction of about three times the finding in Mulago National Referral Hospital [14] 4 years post- introduction of rotavirus vaccine. This reflects a possible and expected effectiveness of rotavirus vaccination in reducing the burden of rotavirus gastroenteritis [21]. The study findings are similar to that observed in Nairobi Kenya (15.2%) 5 years post vaccination rollout [22]. Possibly due to effectiveness of the rotavirus vaccine 5 years post introduction in both countries. Additionally, the studies from both countries were carried out in urban settings which fairly have better environmental hygiene compared to the rural settings. Higher prevalence was seen in; Tanzania (Moshi) 26.4% [23], possibly due to large sample size, slightly longer study duration (5 months) and also the study was carried out in four different facilities therefore making a larger coverage and increasing the chance of rotavirus diarrhea.

Rotavirus diarrhea is one of the most leading causes of dehydration among all the diarrhea causing organisms. In the present study some dehydration and severe dehydration accounted for 71.5% of the participants with rotavirus gastroenteritis in comparison to those who were rotavirus negative, with some and severe dehydration accounting for 24.9%. This is due to increased loss of water and electrolytes without adequate replacement [9]. Because of diarrhea, loss of appetite, vomiting and fever associated with rotavirus, fluid replacement is impaired as fluid loss worsens [24]. The study findings were lower than that observed in a cross sectional study in Bandung, Indonesia where some dehydration and severe dehydration contributed 82.8% [25]. This could be because the study participants were children ≤ 6 months of age who are highly susceptible to fluid loss. This explains the urgent need to intervene in infants with rotavirus diarrhea with zinc and ORS to prevent the progression to dehydration since more than 1.5 million cases of rotavirus diarrhea become severe enough requiring admission and intravenous fluid management [7, 26].

In this study, factors that were found to be significantly associated with rotavirus diarrhea at multivariable level were, child’s age, gender, having another child with diarrhea at home and getting water for domestic use from a well. A child aged less than 12 months was 8.87 times more likely to have rotavirus compared to one older than 12 months. Our study agreed with another study in Uganda and one in Tanzania that there is a higher risk of rotavirus among children less than 12 months compared to those above [15, 27] with prevalence of 61.1% and 61.6% for children less than 12 months respectively. This could be because within this age group, the children are within the oral phase of development and everything they find in their environment is put to the mouth thus putting them at a more risk of acquiring rotavirus diarrhea compared to their other counterparts who are older than 12 months [28]. Also, it could be possible that at this age group, high odds could be related to waning passive immunity from the mother around the time for weaning.

According to this study, a male child was 0.083 times less likely to have rotavirus infection compared to a female one. There are conflicting data on the correlation of rotavirus and gender as some studies show no gender predilection for rotavirus as seen in Baghdad [29] and Northwestern Angola [30]. Other reports such as a study in Mulago Regional Referral Hospital and another in Nigeria found that males were more at risk of having rotavirus diarrhea compared to the females [15, 31]. Further investigation is required in determining the sex variation in rotavirus infection.

A child from a home with another person with diarrhea was 17.82 times more likely to have rotavirus infection compared to a child from a family where there was no one having diarrhea. This also is in agreement with a study carried out in Maiduguri teaching hospital, Borno state in Nigeria, which found that the source of water supply and presence of persons with gastroenteritis in the household were risk factors for infection acquisition with a statistical significance *P* < 0.05 [32]. A similar study in Mwanza, Tanzania also found that living next door to a child with diarrhea as being highly associated with rotavirus infection (*p* = 0.036) [33]. This can be explained by the fact that rotavirus is an infectious disease that is transmitted through the feco–oral route. This means that close contact plays a big role in the transmission of the infection.

This study also noted that a child from a home where water for home use was obtained from a well was 50.17 times more likely to have rotavirus infection compared to one from a home where water was obtained from a tap. This finding was also in line with a finding from a study in Nigeria about the risk factors associated with rotavirus gastroenteritis among under five children where source of water was also associated with rotavirus gastroenteritis with a *P* < 0.05 [32]. Another study finding that concurs with our findings is that in Pader district northern Uganda, where use of unprotected water sources such as wells contributed significantly to watery diarrhea in children less than 5 years, *P* < 0.001 [34]. In this study however causation was not determined but since it is the most common cause of diarrhea it could have been a key contributor to the diarrhea. This could be explained by the fact that rotavirus diarrhea is transmitted orofecally therefore use of water for domestic use from unprotected sources such as wells may carry rotavirus into the water from either animal or human excreta, therefore leading to acquisition of infection if the water is not well treated or boiled.

It is important to note that 15% of the children reported as completely vaccinated had rotavirus and though the explanation for this is not clear, it is possible that some of these children could have been miss classified as having completed rotavirus vaccination give that some mothers/caretakers did not have the vaccination card and the information on the vaccination status was obtained by verbally asking the mother who replied in accordance to what they recall which could have been affected by recall bias.

### Limitations of the study

The study was conducted in a hospital setting, this might not reflect the true burden in the community. However, the researcher also included participants who were attending care as outpatients. The study was carried out within a short period of 3 months therefore the season variations of rotavirus could have affected the study findings. Nutritional status was not studied as an independent factor. The study used an immunochromatographic assay as a rapid diagnostic test which is not the gold standard as compared to RT-PCR. There is a possibility that some of the children documented as having been fully vaccinated could have been wrongly classified since some caretakers/mothers did not have their Childs’ immunization card, and only verbally informed about the immunization status. More so, during data collection, we did not indicate which information about vaccination was card-verified.

### Strength of the study

This was the first study to describe the prevalence of rotavirus diarrhea post introduction of rotavirus vaccination in Fort-portal and probably in Uganda.

## Conclusion

The prevalence of rotavirus diarrhea was high in the post rotavirus vaccination period.

Majority of the participants with rotavirus diarrhea had some dehydration. The factors that were significantly associated with rotavirus diarrhea were child’s age, gender, having another person at home with diarrhea and use of water from the well for domestic purpose. This study demonstrates the need for provision of safe water sources and encouraging treating water for domestic use in the communities in order to reduce the cases of rotavirus.

There is need to carry out more surveillance to find out the possible cause of the diarrhea cases that were seen other than rotavirus.

Given the dehydration distribution with a substantial fraction having ‘no’ dehydration, this suggests that the trigger for hospital referral for diarrhoea is low. Any future study of this subject could include questions regarding distance travelled to reach the hospital or be supplemented by a community health utilization survey (referral behavior relative to the signs/symptoms of diarrhoea and dehydration).

## Data Availability

Data is available upon request. Requests should be sent to lakergoretty890@gmail.com (GL).

## Abbreviations

ORS: Oral Rehydration Salts
FRRH: FortPortal Regional Referral Hospital
